# 

**Published:** 1915-08-28

**Authors:** 


### Personalia.

Sir John Collie has been appointed to be temporary hon. major in the Royal Army Medical Corps, the appointment dating from July 30.

Miss Eleanor S. Hill, M.B., B.S. (Lond.), has been appointed resident medical officer at the Manchester Children's Hospital, Pendlebury, and takes up her duties on September 1.

Rates of Subscription: Twelve months, 6/6; Six months, 4/-; Three months, 2/-, post free to any address in the United Kingdom. Abroad, Twelve months, 8/8; Six months, 5/-; Three months, 2/3, post free.—Address The Subscription Dept., "The Hospital," The Hospital Building, 28/29 Southampton Street, Strand, London, W.C.

